# Effect of Adjuvant Mitomycin-C on Recurrent Rhegmatogenous Retinal Detachment With Proliferative Vitreoretinopathy Managed by Relaxing Retinotomy and Retinectomy

**DOI:** 10.1155/joph/9927416

**Published:** 2025-03-19

**Authors:** İhsan Gökhan Gürelik, Hüseyin Baran Özdemir, Beste Gizem Köse, Ahmet Burak Acar

**Affiliations:** Department of Ophthalmology, Gazi University Faculty of Medicine, Ankara, Turkey

**Keywords:** mitomycin-C, proliferative vitreoretinopathy, recurrent retinal detachment, retinectomy, retinotomy, rhegmatogenous retinal detachment, vitrectomy

## Abstract

**Purpose:** To evaluate the role of adjuvant mitomycin-C (MMC) use in cases of recurrent rhegmatogenous retinal detachment (RRD) complicated by proliferative vitreoretinopathy (PVR) managed by relaxing retinotomy and retinectomy (RR).

**Methods:** A retrospective analysis of consecutive patients undergoing vitreoretinal surgery with RR for RD and PVR was conducted. Patients were divided into two groups: those receiving 20 μg/0.1 mL MMC via the MMC sandwich method (Group 1) and those who did not (Group 2).Demographics, surgical characteristics, visual outcomes, and complications that may related to MMC were analysed.

**Results:** A total of 28 patients (14 eyes per group) were included in the study. Differences in baseline variables between groups were not significant (*p* > 0.05). The mean follow-up was 15.2 ± 12.2 months. In Group 1, mean preoperative best-corrected visual acuity (BCVA) improved from 2.72 ± 0.70 logMAR to 1.59 ± 0.61 logMAR postoperatively (*p*=0.001). In Group 2, mean preoperative BCVA increased from 2.06 ± 0.80 logMAR to 1.77 ± 0.94 logMAR (*p*=0.261). Re-surgery rates were significantly lower in Group 1 (21.4%) than in Group 2 (92.8%, *p*=0.001). Final retinal attachment was achieved in 100% of eyes in both groups. Postoperative mean intraocular pressure (IOP) was 16.29 ± 4.46 mmHg in Group 1 and 13.92 ± 1.44 mmHg in Group 2 (*p*=0.081). No MMC-related toxicity was observed clinically.

**Conclusions:** MMC, applied via the sandwich technique, appears safe and is associated with high anatomical and functional success rates while reducing re-operations.

**Summary**

• **What is already known on this topic:** Relaxing RR for PVR effectively relieve traction, but re-proliferation, particularly at the retinotomy margins, can compromise surgical success.
• **What this study adds:** This study demonstrates that adjuvant MMC may prevent PVR progression in recurrent retinal detachment cases following RRD, enhancing surgical outcomes.
• **How this study might affect research, practice or policy:** Our findings suggest that MMC can be safely utilized in conjunction with relaxing RR offers a safe and effective strategy for managing recurrent retinal detachment with PVR, potentially reducing re-operations and informing future treatment protocols.

## 1. Introduction

Proliferative vitreoretinopathy (PVR) is an abnormal wound-healing response and is the most common cause of recurrence after rhegmatogenous retinal detachment (RRD) [1]. It is seen in 5%–10% of cases after vitreoretinal surgical procedures for RDD [2]. The development of PVR after vitreoretinal surgery for RRD is usually seen between 4 and 12 weeks [3, 4]. Intraocular inflammation, preoperative, intraoperative or postoperative haemorrhage, aphakia, large retinal tear and detachment area, choroidal detachment, hypotony, excessive cryotherapy and endolaser application are factors that may increase the risk of PVR formation [1, 2]. The success rate of recurrent surgeries has been reported to be between 60% and 75% in patients with recurrent detachment due to the development of PVR [2].

Various surgical methods have been tried for the treatment of PVR after surgery for RRD. PVR causes tractional membranes which may occur in the anterior-posterior, perpendicular or circumferential direction with retinal shortening and membrane contraction. In the current surgical approach, PPV is applied in combination with scleral buckle or alone. Advanced vitreoretinal surgical techniques such as membrane peeling, relaxing retinotomy and retinectomy (RR) are performed during surgery [5]. In various series, final anatomical success rates have been reported between 51% and 90% in patients who underwent retinectomy [6]. Anatomical success rate was found to be higher in patients who developed relapse due to PVR and underwent RR procedures compared to patients who did not undergo this procedure [7]. However, these retinectomy areas can lead to new PVR formations and recurrent detachments.

Various studies have been done to prevent the development of PVR on many molecules such as methotrexate, 5-fluorouracil (5-FU), triamcinolone acetonide but none of them are effective on PVR management [8–10]. To reduce the development of PVR, mitomycin-C (MMC) has been used successfully in patients with traumatic RD and proliferative diabetic retinopathy without complications [11–13]. There is no study in the literature about the effectiveness of MMC in cases of recurrent RD due to PVR after RRD.

In this study, we aimed to determine the role of adjuvant MMC use with the novel MMC sandwich method in cases of recurrent RD caused by PVR after RRD.

## 2. Materials and Methods

In the present study, we retrospectively evaluated the medical records of patients with recurrent RD due to PVR after vitrectomy for RRD who underwent vitreoretinal surgery with RR more than one quadrant (90°) between 2019 and 2022. All the patients had RR extending more than one quadrant, PVR C or more and varying degrees of retinal shortening that could require RR. Patients who had undergone ocular surgery before vitrectomy for RRD (except uncomplicated cataract surgery) and had a hereditary vitreoretinal disease, inadequate follow-up (less than 6 months), and incomplete data were excluded. Patients' data were divided into two categories: MMC used (Group 1) and unused (Group 2). Twenty-eight eyes of 28 patients, including 14 with MMC and 14 without MMC, were included in the study. Written informed consent was obtained from all patients before every surgical procedure. The Ethics Committee of Gazi University approved the study protocol (approval no: 2022-1162). The study was in accordance with the principles of the Declaration of Helsinki. Patients or the public were not involved in the design, or conduct, or reporting, or dissemination plans of our research.

All patients underwent detailed ophthalmic examination, including, best-corrected visual acuity (BCVA), age, gender, history, preoperative intraocular pressure (IOP), lens status, the extent of RD, the extent of RR, applied surgical interventions were evaluated. Previous surgical history was recorded, including the type of prior procedures (vitrectomy alone, combined scleral buckle and vitrectomy, lensectomy with or without IOL implantation) and intraocular tamponade agents used (gas or silicone oil). The PVR was graded intraoperatively according to the Retina Society Classification System [14]. All patients underwent a comprehensive ophthalmologic examination at all visits. The patients were evaluated on day 1, weeks 1 and 2, month 1, and every 1–3 months during follow-up. All patients were also documented with colour fundus photographs, anterior segment photographs, macular optical coherence tomography (OCT), and retinal nerve fibre layer (RNFL) thickness scans (Spectralis, Heidelberg, Germany) at all visits except postoperative day 1.

Postoperative BCVA, IOP, retinal re-attachment, final anatomic and visual outcomes, and postoperative complications were evaluated. Final anatomical success was considered retinal attachment at least 6 months. Previously described potential toxicity of intraocular MMC, such as intraocular inflammation, ciliary body atrophy and hypotonia, optic atrophy, macular atrophy and retinal vascular occlusion, were investigated postoperatively [15]. OCT and RNFL thickness scans were used to screen for possible retinal toxicity. IOP was used to screen for possible toxicity to the ciliary epithelium. Hypotony was accepted as an IOP less than six mmHg.

### 2.1. Surgical Technique

The MMC sandwich technique is used to prevent the development of PVR and has been described before [11]. A preclinical study has reported the use of the MMC sandwich technique at concentrations up to 20 μg/0.1 mL MMC without causing toxicity to vital intraocular structures [16]. All procedures were performed by two experienced vitreoretinal surgeons (IGG and HBO) using 25-gauge instrumentation (Alcon Laboratories, Inc., Fort Worth, TX). Scleral buckling was not utilized in any case. After advanced vitreoretinal surgical procedures such as phacoemulsification in cases with significant lens opacity, pars plana vitrectomy, membrane peeling, internal limiting membrane peeling (if not performed before), and relaxing RR, the retina was attached with fluid-air exchange. Perfluorocarbon liquid (PFCL) was injected until covering the posterior vital structures, including the optic disc and macula, to the border of RR sites, and retinal breaks. Ciliary epithelium and other anterior segment structures were protected from MMC contact with air filling the rest of the eye. Then, a 20 μg/0.1 mL concentration of MMC solution (dyed with 0.05 cc Brillant Blue to make it visible) was carefully injected above the PFCL bubble until it covered the major vascular arcade, proliferating sites and potential areas of PVR development such as RR and retinal tears. MMC was removed after 60 s. Finally, the remaining PFCL was removed, and all eyes were filled with 5000 cs silicone oil after endolaser. The patients were followed for at least 6 months.

### 2.2. Statistical Analysis

The data were analysed using SPSS version 22.0 (IBM SPSS Statistics, Armonk, NY, USA). A significance level of 0.05 was set for all analyses. The significance of the preoperative and postoperative changes within each group was tested using the Wilcoxon signed ranks test. Mann–Whitney *U* tests were performed to test the significance of group differences in baseline variables. The significance of group differences was assessed using the chi-square test and Fisher's exact test for categorical variables.

## 3. Results

Twenty-eight eyes of 28 (22 male, four female) recurrent RD patients with PVR requiring more than 90° RR were included in the study. The mean follow-up time was 15.2 ± 12.2 months (IQE 8–17 months). There was no statistical difference in preoperative characteristics such as gender, age, preoperative IOP, visual acuity, macular attachment status, RD extension, PVR severity, lens status, mean time since previous surgery and mean number of previous surgeries. No significant differences were found in the type or frequency of previous surgeries or tamponade agents between groups (all *p* > 0.05). Baseline demographics and characteristics are summarized in Table 1.

Surgical procedures and outcomes are listed in Table 2. In both groups, 90° to 360° RR was performed with advanced VRC techniques, and there was no difference in RR extension (*p* :  0.815). There was a statistically significant increase in Group 1 (*p*=0.001), when preoperative BCVA (2.72 ± 0.70 logMAR) was compared with final postoperative BCVA (1.59 ± 0.61 logMAR). Although postoperative BCVA (1.77 ± 0.94 logMAR) increased in Group 2 compared to preoperative BCVA (2.06 ± 0.80 logMAR), this increase was not statistically significant (*p*=0.261). Postoperative BCVA in Group 1 was higher than in Group 2, but this difference was not statistically significant (*p*=0.550). The mean duration of silicone oil tamponade was 5.89 ± 2.73 (2–10) months in Group 1 and 5.33 ± 1.71 (2–7) months in Group 2 (*p*=0.436). Final lens status showed 1/12/1 phakic/pseudophakic/aphakic eyes in Group 1 and 1/11/2 in Group 2 (*p*=0.828). The final retinal attachment rate and silicone oil removal rate were 100% in both groups. The mean number of surgeries in Group 1 (1.21 ± 0.57) was lower than in Group 2 (1.93 ± 0.73) and this difference was statistically significant (*p*=0.008). Success with single surgery in patients with recurrent RD was obtained 13 of 14 patients (92.8%) in Group 1 and three of 14 patients (21.4%) in Group 2 (*p*=0.001). Recurrent RD occurred in eyes in both groups due to re-proliferation and PVR under silicone oil. At the final examination, the mean IOP was 16.29 ± 4.46 mmHg in Group 1 and 13.92 ± 1.44 mmHg in Group 2 (*p*=0.081). There was no acute or accelerated macular or RNFL atrophy in both groups by OCT/RNFL thickness measurements. Postoperative final OCT measurements showed peripapillary RNFL thickness of 86.00 ± 34.45 μm in Group 1 and 62.79 ± 19.37 μm in Group 2 (*p*=0.062), and central macular thickness of 277.36 ± 94.38 and 221.79 ± 68.54 μm (*p*=0.137).

## 4. Discussion

PVR is the most important and common cause of recurrence in patients with RRD. Despite developing surgical techniques, conditions such as time until surgery, age, intraocular inflammation, and haemorrhage increase the risk of PVR and reduce surgical success. The advancement of vitrectomy techniques and instruments has led to improved surgical success rates in PVR. The incorporation of advanced techniques have raised the success rate to 60%–75% [2]. Despite these enhancements, over 25% of initially successful cases experience re-detachment due to recurrent retinal traction. Moreover, visual outcomes are less than optimal, with only 40%–80% of cases achieving ambulatory vision (5/200 or better) despite achieving anatomical success [17].

Successful management of PVR necessitates a complete PPV and precise membrane peeling. Nevertheless, the persistence of traction or retinal shortening can jeopardize the surgical outcomes. In such cases, achieving anatomical success often involves the implementation of RR. This procedure entails incising the contracted retina, typically in the peripheral inferior region, to facilitate the attachment of the rest of the retina. The use of RR has been recognized as a successful surgical approach in vitrectomy for eyes with advanced PVR associated with RD. Recent series on circumferential RR surgery have reported initial anatomical success rates ranging from 51.3% to 76.7% [6, 18–20]. Although surgical success could be increased with RR, re-proliferation of membranes at the posterior edge of the RR cause recurrent RD has been recently reported in 13% of eyes with inferior retinectomy and in 23% of eyes with combined radial retinotomy and circumferential retinectomy [5, 21, 22].

Various studies have been conducted to prevent PVR development and increase the surgical success through adjuvant therapies, including oral prednisolone, intravitreal triamcinolone acetonide, dexamethasone, low molecular weight heparin, 5-FU, daunorubicin, intravitreal anti-VEGF agents, oral isotretinoin and methotrexate [1]. Despite promising results in preclinical studies of many agents, their effectiveness has yet to be shown in large prospective human clinical trials.

MMC has been used for many years in ophthalmic procedures such as trabeculectomy, pterygium surgery, and photorefractive keratectomy [23]. It is recognized for MMC's capability to diminish the primary fibrovascular proliferation response, which constitutes the principal mechanism in the development of PVR. This impact has been validated through in vitro studies and experimental PVR models, specifically on retinal pigment epithelium (RPE) cells [24]. Although the effect of MMC on cell proliferation has long been known, concerns about its potential toxicity to posterior segment structures prevented its use in retinal surgeries until recent years. Advances in imaging technologies and surgical techniques have now brought about research into clinical applications [11, 12]. Assi et al. applied a soaked sponge from the sclerotomy area to the air-filled vitreous and the chorioretinal wound site in patients with traumatic RD [12]. In our previous study, we prevented MMC contact and toxicity from the posterior segment structures with PFCL and anterior segment structures with the air filling the rest of the eye, defined as the ‘MMC sandwich technique', and PVR was successfully prevented in all eyes with traumatic RD [11].

In the present study, it has been shown that MMC application reduces repeat surgeries in recurrent RD patients compared to control group. The final functional outcomes were found significantly higher with adjuvant MMC use although final anatomical outcomes were similar.

In our study, RR was performed in advanced PVR (PVR C and D) eyes during vitrectomy. Our primary success rate with MMC sandwich method was 92.8% and final success rate was 100%. Zand et al. reported successful retinal attachment as the initial anatomical outcome of 73.8% after the first retinotomy surgery in patients similar with our study [6]. Frenkel et al. was achieved primary anatomical success in 76.7% of patient underwent RR [20]. In our study, the high success rate in the first surgery suggests that it is related to the use of adjuvant MMC reducing rate of recurrent PVR formation. Conversely, the low single surgery success rate in Group 2 (21.4%) contrasts with reported rates of 51.3%–76.7% in recent series [6, 18–20]. This may reflect greater PVR severity, differences in patient selection, or the absence of MMC, though our cohort's complexity (e.g., extensive RD and advanced PVR grades) likely contributed. Recurrent RD in Group 2 predominantly occurred due to re-proliferation under silicone oil, highlighting the challenge of managing PVR without adjuvant therapy.”

Our findings revealed a significant improvement in BCVA at the last follow-up compared to the baseline in MMC used group. Idrees, Sridhar, and Kuriyan indicated that multiple surgeries in patients with PVR are associated with worse visual outcomes [1]. The significant reduction in reoperation rates with the use of MMC may have maintained the increase in BCVA in our study.

It is known that in patients performed RR may lead to absorption of intraocular fluid and subsequent ocular hypotony with bare RPE. In our study, there was no newly developed hypotony after the surgery in both groups. This finding also showed that MMC sandwich method is a safe technique to prevent possible intraocular toxicity related to MMC. The absence of MMC-related toxicity, as assessed by stable 1OP and clinical exams, aligns with preclinical data [16]. However, while OCT/RNFL scans showed similar measurements between groups, the retrospective nature of our study limits definitive attribution of structural changes to MMC versus PVR progression. Future studies with detailed OCT metrics are needed to confirm safety.

Limitations of our study are its retrospective nature and the low number of patients. Future prospective randomized studies are needed to evaluate the effect of MMC in PVR development in patients with recurrent RD.

In conclusion, the use of intraoperative adjuvant MMC with MMC sandwich technique, during advanced vitreoretinal manoeuvres and RR, safely reduces the re-proliferation of membranes and repeat surgeries with favourable functional outcomes in patients with recurrent RD caused by PVR after RRD.

## Figures and Tables

**Table 1:** Characteristics of patients.

	Group 1 (*n*: 14)	Group 2 (*n*: 14)	*p* value
Age	49.07 ± 20.95 (10–72)	53.29 ± 24.90 (6–86)	0.541
Gender (M/F)	11/3	9/5	0.339
Pre-operative BCVA	2.72 ± 0.70 logMAR (1.50–3.50)	2.06 ± 0.80 logMAR (1.00–4.00)	0.061
Pre-operative IOP	12.71 ± 4.46 mmHg (4–21)	14.38 ± 2.36 mmHg (10–20)	0.141
Pre-operative lens status			
Phakic	4 (28.6%)	3 (22.4%)	
Pseudophakic	9 (64.3%)	9 (69.2%)	
Aphakic	1 (7.1%)	2 (15.4%)	0.617
Prior surgery			1.000
Vitrectomy alone	12 (85.7%)	12 (85.7%)	
Vitrectomy with scleral buckle	2 (14.3%)	2 (14.3%)	
Prior surgery tamponade			0.241
C3F8	0	2 (14.3%)	
Silicone oil	14 (100%)	12 (85.7%)	
Macula			0.615
Macula on	4 (28.6%)	4 (30.8%)	
Macula off	10 (71.4%)	9 (69.2%)	
The extend of RD			0.193
One quadrant	3 (21.4%)	1 (7.7%)	
Two quadrants	3 (21.4%)	8 (61.5%)	
Three quadrants	3 (21.4%)	2 (15.4%)	
Total RD	5 (35.7%)	2 (15.4%)	
PVR			0.087
PVR C1	6 (42.9%)	2 (15.4%)	
PVR C2	4 (28.6%)	9 (69.2%)	
PVR C3	1 (7.1%)	0	
PVR D1	2 (14.3%)	1 (7.7%)	
PVR D2	1 (7.1%)	1 (7.7%)	
Time since previous surgery	45.00 ± 18.08 days (20–75)	41.43 ± 15.98 days (20–60)	0.585

Abbreviations: BCVA, best-corrected visual acuity; IOP, intraocular pressure; PVR, proliferative vitreoretinopathy; RD, retinal detachment.

**Table 2:** Surgical characteristics and postoperative outcomes.

	Group 1 (*n*: 14)	Group 2 (*n*: 14)	*p* value
Phacoemulsification + IOL impl.	3 (21.4%)	0	0.111
The extent of retinectomy			0.815
One quadrant	2 (14.3%)	3 (21.4%)	
Two quadrants	6 (42.9%)	7 (50.0%)	
Three quadrants	4 (28.6%)	2 (14.3%)	
360 degree	2 (14.3%)	2 (14.3%)	
Success with single surgery for recurrent RD (except silicone oil removal)	13 (92.8%)	3 (21.4%)	**0.001**
Mean surgery number, except silicone oil removal (min–max)	1.21 ± 0.57 (1–3)	1.93 ± 0.73 (1–4)	**0.008**
Mean duration of silicone oil (min–max)	5.89 ± 2.73 (2–10) months	5.33 ± 1.71 (2–7) months	0.436
Final lens status			
Phakic	1 (7.1%)	1 (7.1%)	
Pseudophakic	12 (85.7%)	11 (78.6%)	
Aphakic	1 (7.1%)	2 (14.3%)	0.828
Final BCVA	1.59 ± 0.61 logMAR (0.60–3.00 logMAR)	1.77 ± 0.94 logMAR (0.30–3.00 logMAR)	0.550
Final IOP	16.29 ± 4.46 mmHg (10–26 mmHg)	13.92 ± 1.44 mmHg (11–16 mmHg)	0.081

*Note:* Bold values are statistically significant.

Abbreviations: BCVA, best-corrected visual acuity; IOL impl, intraocular lens implantation; IOP, intraocular pressure; RD, retinal detachment.

## Data Availability

The data that support the findings of this study are available from the corresponding author upon reasonable request.
